# Influence of Miscibility Phenomenon on Crystalline Polymorph Transition in Poly(Vinylidene Fluoride)/Acrylic Rubber/Clay Nanocomposite Hybrid

**DOI:** 10.1371/journal.pone.0088715

**Published:** 2014-02-14

**Authors:** Mohammad Mahdi Abolhasani, Minoo Naebe, Azam Jalali-Arani, Qipeng Guo

**Affiliations:** 1 Chemical Engineering Department, Kashan University, Kashan, Iran; 2 Institute for Frontier Materials, Deakin University, Geelong, Australia; 3 Department of Polymer Engineering and Color Technology, Amirkabir University of Technology, Tehran, Iran; University of Nebraska-Lincoln, United States of America

## Abstract

In this paper, intercalation of nanoclay in the miscible polymer blend of poly(vinylidene fluoride) (PVDF) and acrylic rubber(ACM) was studied. X-ray diffraction was used to investigate the formation of nanoscale polymer blend/clay hybrid. Infrared spectroscopy and X-ray analysis revealed the coexistence of β and γ crystalline forms in PVDF/Clay nanocomposite while α crystalline form was found to be dominant in PVDF/ACM/Clay miscible hybrids. Flory-Huggins interaction parameter (B) was used to further explain the miscibility phenomenon observed. The B parameter was determined by combining the melting point depression and the binary interaction model. The estimated B values for the ternary PVDF/ACM/Clay and PVDF/ACM pairs were all negative, showing both proper intercalation of the polymer melt into the nanoclay galleries and the good miscibility of PVDF and ACM blend. The B value for the PVDF/ACM blend was almost the same as that measured for the PVDF/ACM/Clay hybrid, suggesting that PVDF chains in nanocomposite hybrids interact with ACM chains and that nanoclay in hybrid systems is wrapped by ACM molecules.

## Introduction

Over the last decade, polymer nanocomposites with organically modified layered silicates have received much attention due to their high temperature stability, improved mechanical and barrier properties [1]–[4]. Dispersion of a few precents of clay in the polymer matrix has been carried out by direct adsorption and impregnation [5]. Due to the small enthalpy of mixing polymer and layered silicates, the surfaces of silicate layers have been modified to improve dispersion within polymer matrix [6]–[11]. Although the increased entropy offers the driving force for the physical adsorption process, the direct adsorption of uncharged polymers onto a clay surface appears unlikely due to the small enthalpy variation of this process. Consequently, the specific interactions driven by hydrogen bonding and/or dipole-dipole interaction play an important role to increase both the regular dispersion of clay platelets and the change in crystalline polymorphs of polymers.

Recently, PVDF/ACM blends have been studied [12]–[18]. In our previous papers [16]–[18], we have investigated various aspects of miscibility and crystallization behaviours for poly(vinylidene fluoride) (PVDF) and acrylic rubber(ACM) systems. We showed that PVDF and ACM are miscible in ACM rich blends and partially miscible in the blends with more than 50 wt% PVDF. This phenomenon is due to specific interaction between CF2 group of PVDF and carbonyl group of ACM. The main advantage of this blend is the ability of PVDF to crystalize even in ACM rich blends contrary to many other miscible blends containing PVDF [16], [18].

Much effort has been focused on the development of β and/or γ polymorphs in the presence of organically modified layered silicates in PVDF [19]–[27] and different mechanisms have been proposed to describe this phenomenon [25]–[27]. However, to the best of our knowledge the effects of miscible amorphous component on the crystalline structure and formation of different polymorphs in the PVDF nanocomposite have not been investigated.

In the present study, we report the dispersion of organically modified layered silicates in a miscible polymer blend and investigate the effects of the miscible amorphous polymers on the formation of different polymorphs of PVDF. Wide angle x-ray diffraction (WAXD) and Fourier transform infrared spectroscopy (FTIR) analysis of the nanocomposite hybrids are used to provide information about various polymorphs formations. The interaction in a nanocomposite can be described by the thermodynamic interaction energy density (B) based on the classical Flory-Huggins theory [28]. Differential scanning calorimetry (DSC) is used to determine the B values of the PVDF/ACM/Clay nanocomposite system. This was achieved through combining the binary interaction model and the melting point depression originally proposed to evaluate the specific interaction between two polymers. We have attempted to associate the crystalline structure of PVDF in miscible nanocomposite to B values obtained from binary interaction model.

## Experimental

### Materials and Sample Preparation

PVDF (Kynar 710) MFR of 25 g/10 min (2328C/12.5 kg load) from Arkema and acrylic rubber (Grade AR71) from Zeon Advanced Polymix Co.(Thailand) were used in this work. The major component of the acrylic rubber was poly (ethyl acrylate) (PEA), which contained a minor amount (5%w) of chlorine cure-site monomer. Cloisite 30B is organically modified clay with a cation exchange capacity of 90 meq/100 g, supplied by Southern Clay. All components were dried in a vacuum oven at 80°C for at least 12 h before processing. The nanocomposite with 5%(wt) nanoclay and a different PVDF/ACM ratio (specification and composition in table 1.) were prepared using a Brabender internal mixer at a rotation speed of 100 rpm at 190°C for 10 min. Samples were hot pressed at 200°C to a thin film and allowed to slowly cool down to room temperature. It’s worth noting that in all samples nanoclay was added to miscible PVDF/ACM blends.

**Table 1:** List of samples prepared.

Sample	PVDF(wt%)	ACM(wt%)	Clay(wt%)
NPVDF	100	0	5
N40/60	40	60	5
N30/70	30	70	5
N20/80	20	80	5
N10/90	10	90	5
NACM	0	100	5

### Characterization

Differential scanning calorimetery (DSC) was conducted using a TA Instrument Q200. To measure the equilibrium melting point, all samples were melted at 210°C for 10 min then each cooled down to the desired isothermal temperature and maintained at that temperature until the degree of crystallinity was not increasing any more. After completion of isothermal crystallization the sample subsequently reheated to 210°C at a heating rate of 20°C /min to obtain the melting endotherm curve.

Fourier transform infrared spectroscopy (FTIR) was carried out using Bruker 70 equipped with ATR unit. FTIR spectra were acquired(64 scans at 4 cm^−1^ resolution ) from500 Cm^−1^ to 1500 Cm^−1.^

X-ray diffraction measurement was performed on a Panalytical XRD instrument. The data was recorded in the range of 2θ = 5–40° and 2–10°. Samples were scanned continuously with a 0.5° scan step and 1 second scan time. Optical microscopy (OM) was carried out using polarizing microscopes (Nikon Eclipse 80i) equipped with a CCD camera under the cross polarization state.

The composite samples were sectioned using a Leica UC6 ultramicrotome with FC6 cryochamber at −120°C, at a nominal thickness of 70 to 80 nm. Sections were imaged using a Gatan Orius SC1000 digital camera on a JEOL 2100 transmission electron microscope (TEM) operating at an accelerating voltage of 200 kV.

## Results and Discussion

### Morphology and Clay Dispersion

**Figure 1:**
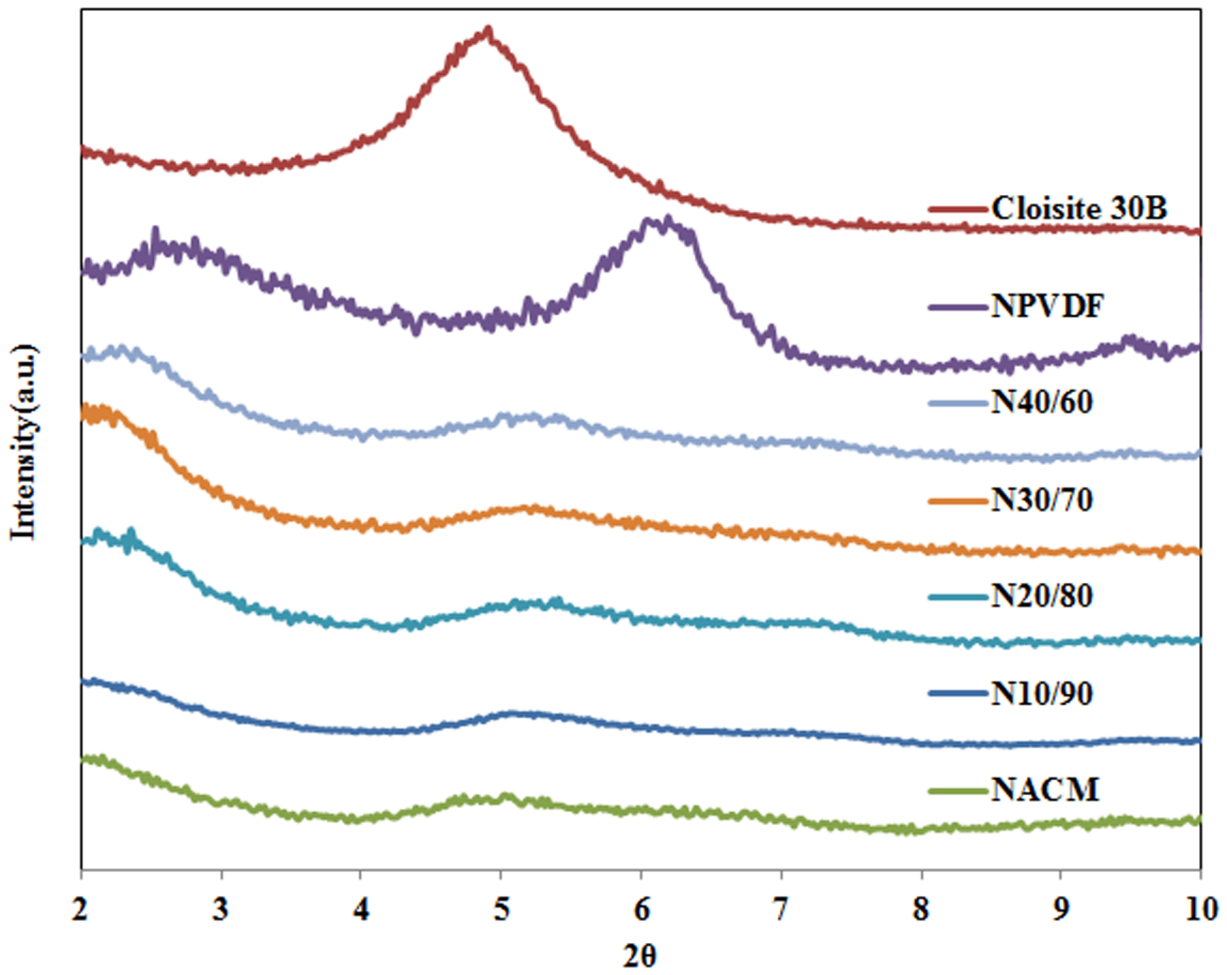
WAXD profile of all prepared samples.

Figure 1 presents the WAXD patterns of samples. The Cloisite 30B has a d-spacing of 1.8 nm, evidenced by the XRD peak at 2θ∼4.8°. In the NPVDF sample containing 5 wt% clay, this peak is shifted towards the left (lower frequencies), resulting in a diffused peak at 2θ∼2.5°, corresponding to d-spacing of 3.4 nm. This suggests that there are some regions in which clay forms an intercalated nanocomposite structure. This type of structure is formed due to either the interaction between the modified clay and PVDF or shear induced intercalation. The peak at 2θ∼5.8° corresponding to the d-spacing 1.4 nm could be due to the second order diffraction d(002) [29]. The appearance of this peak could be attributed to a partially collapsed structure resulting from quaternary ammonium degradation.

NACM sample and samples containing ACM showed different behaviour. These samples all demonstrate two small peak at 2θ∼2° corresponding to d-spacing of 4.2 nm and a broad peak at around 2θ∼5° which is almost the same as neat Cloisite 30B peak. However, the relative intensity of the peak reduced significantly suggesting partial exfoliation of nanoclay. A small fraction of nanoclay in sample containing ACM remains in form of local aggregates.

**Figure 2:**
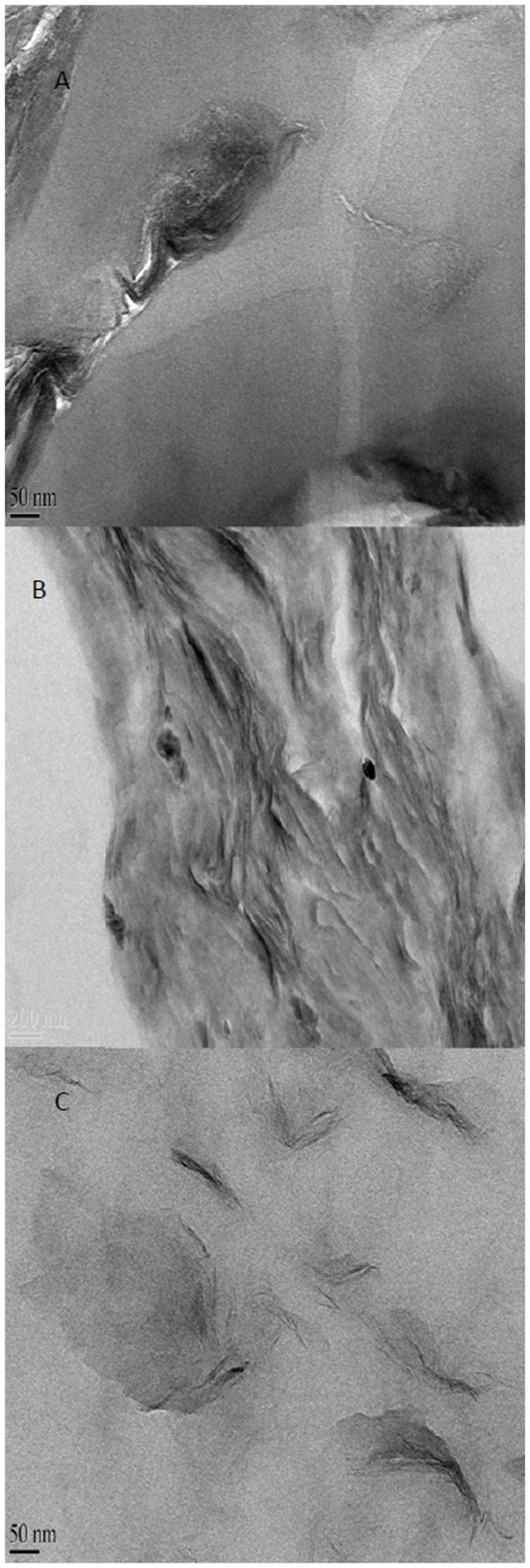
TEM images of (A)NPVDF, (B) NACM and (C)N20/80.

TEM images for NPVDF, NACM and N20/80 sample are shown in Figure 2. Figure 2 (A) clearly shows clay tactoids with a thickness of ∼150 nm in NPVDF sample. This is due to the high interfacial tension. From the TEM images, it is clear that the NPVDF sample failed to form an exfoliated structure. In contrast, TEM images of NACM and N20/80 (Fig. 2 (B, C)) show individual nanoclay layers as well as stacks containing parallel oriented layers with various degree of intercalation. The intercalation of ACM chains between the silicate layers is enhanced by the strong polar interaction developed between the oxygen groups of silicate and the oxygen groups of ACM. Therefore, it can be speculated that ACM has a better affinity to organically modified layered silicates compared with PVDF.

**Figure 3:**
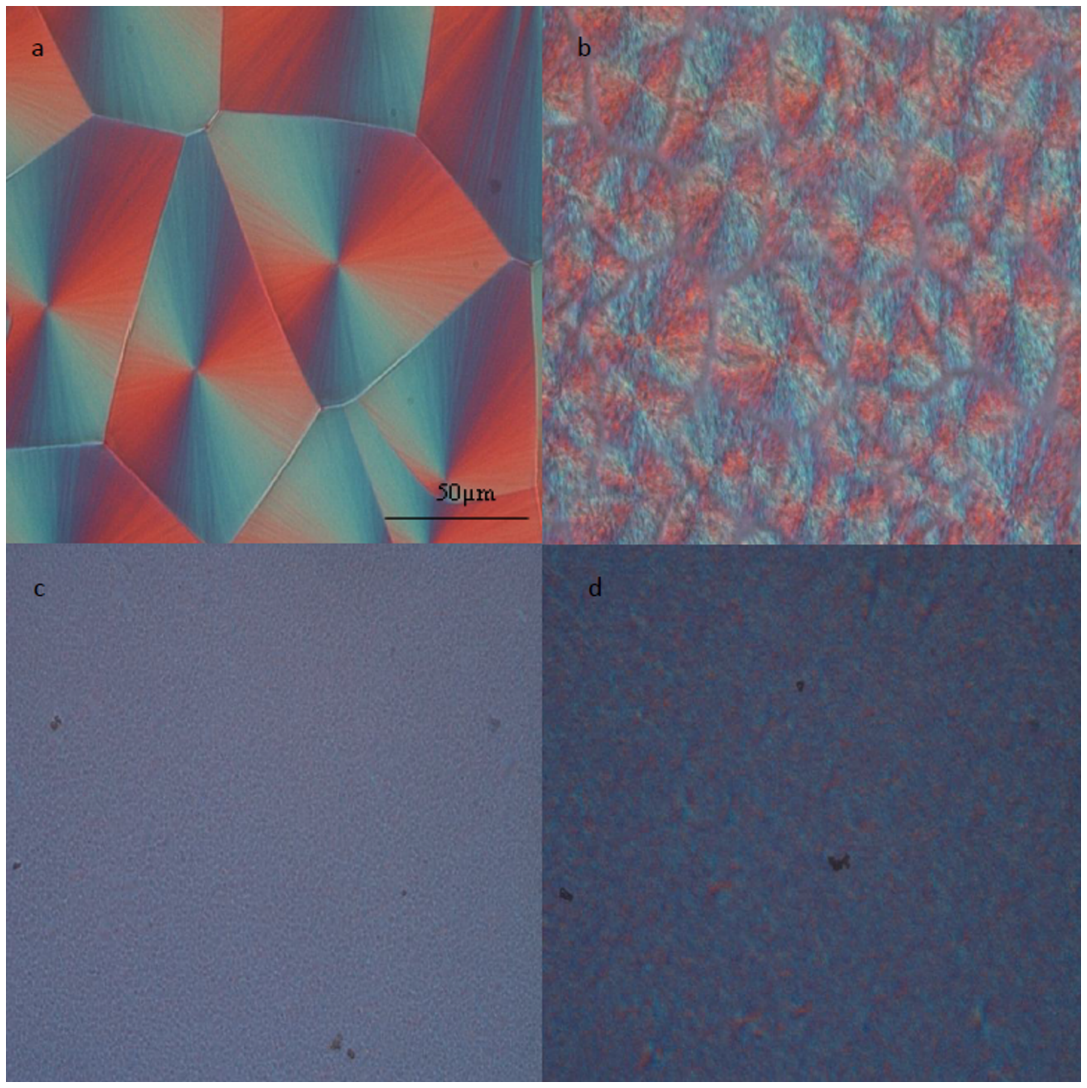
POM images of (A) PVDF, (B) PVDF20/ACM80 blend, (C) NPVDF, and (D) N20/80 samples. Samples were isothermally crystalized at 150°C for one hour.

The nucleation effect of nanoclay could also be illustrated by polarised optical microscopy (POM) images of PVDF, PVDF20/ACM80, NPVDF, and N20/80 samples, as seen in Figure 3 These samples were all isothermally crystalized at 150°C for one hour. Neat PVDF showed typical spherulitic structure which is spherulite crystals with lamellar splay texture displaying a clear Maltese cross extinction pattern under cross polarization. On the other hand, the miscible PVDF20/ACM80 blend formed dendritic spherulite structure with more open texture. By adding the nanoclay into the above samples, the size of spherulites in nanocomposite and nanocomposite hybrid becomes too small to be detected by POM. This can be attributed to the nucleating effect of nanoclay. A large number of nuclei, created from the nucleation agents, simultaneously grow in a restricted space and lead to the small spherulites.

### Crystalline Structure of PVDF/ACM/Clay Hybrids

We demonstrated previously [16]–[18] that the neat PVDF and PVDF/ACM blends formed α polymorph, while PVDF/Clay nanocomposite induce both β and γ polymorphs. In this paper our aim is to investigate the crystalline structure of PVDF in miscible hybrid of PVDF/ACM/Clay.

**Figure 4:**
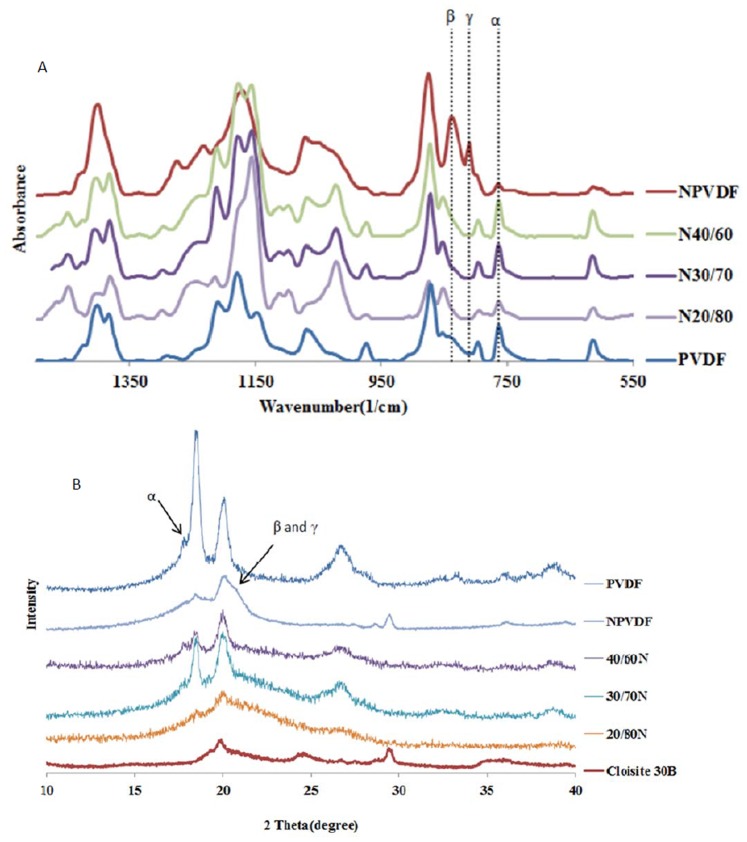
(A) WAXD pattern of neat PVDF and nanocomposite hybrids showed α polymorph characteristic peak while PVDF nanocomposite demonstrated β and γ phase (B) FTIR spectrum of samples showed the same results as WAXD.

Figure 4 (A) presents the WAXD patterns of neat PVDF, NPVDF and nanocomposite hybrids. The three α phase peaks of neat PVDF in WAXD plot observed at 2θ∼17.7°, 18.4° and 20.0° correspond to the (100), (020) and (110) planes and d-spacings of 5.08, 4.88 and 4.52 Å, respectively. Nevertheless, according to the literature the only specific peak of α phase is 2θ∼17.7° and peaks at 2θ∼ 18.4° and 20.0° overlap with β and γ characteristic peaks [14]. As for the NPVDF sample, a shoulder in the right hand peak of 20.0° is observable. The peak at 2θ∼20.7° corresponds to d-spacing 0.427 nm for β phase. γ phase PVDF has a very similar d-spacing reflection at 0.431 nm [22]. Therefore, WAXD pattern suggests the formation of β and/or γ phases. However, the characteristic peak of α phase (2θ∼17.7°) disappears and is no longer present. This means that nanoclay hindered the formation of α polymorph while β and γ phase peaks have overlapped. Interestingly, PVDF/ACM/Clay hybrids again showed α characteristic peaks at 2θ∼17.7°, 18.4° and 20.0°. It seems that PVDF crystalline structure in miscible nanocomposite samples is completely different with that of NPVDF sample.

To further clarify the formation of β and/or γ phase in NPVDF sample and its phase transformation to α polymorph in nanocomposite hybrids, FTIR technique was used to investigate the skeletal and chain conformational changes of PVDF segments. Figure 4 (B) displays the FTIR spectra of neat PVDF, NPVDF and miscible nanocomposite hybrids. The frequencies and the vibrational assignments for α, β and γ phases are 763, 811 and 839 cm^−1^, respectively [27]. Neat PVDF and nanocomposite hybrids show only α phase characteristic peak, while NPVDF sample showed both β and γ phase peak. This observation supports the WAXD study findings, demonstrating that the presence of nanoclay hindered the formation of α polymorph in NPVDF sample while miscibility induced α phase in hybrids nanocomposites. Therefore, α → (β, γ) → α transitions occurred from neat PVDF to PVDF nanocomposite and to the miscible nanocomposite hybrids. Formation of β and γ polymorph in the NPVDF sample can be attributed to similar crystal lattices between clay and the β polymorph [26], various velocity regimes in nanocomposites [25] and the presence of an ion-dipole interaction between nanoclay layers and PVDF chains in molten state [27], while in miscible nanocomposite hybrids there is no interaction between PVDF and clay, causing ACM to act like a compatibilizer between PVDF and clay platelets.

In other words, it can be speculated that PVDF chains in nanocomposite hybrids experience the same surroundings as PVDF chains in PVDF/ACM blends, therefore all PVDF chains are in the same velocity regime. To verify the above speculation we have determined the interaction parameter for nanocomposite hybrids and compared it with PVDF/ACM blends.

### Melting Point Depression and Interaction Parameter Determination

The evaluation of the specific interaction between the crystallizable polymer chains and its surrounding can be made by combining the melting point depression and the binary interaction model for heat of mixing. The relation between the melting point depression and the interaction energy parameter in the mixture can be described by the following equation [30]:
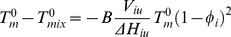
(1)where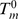 and 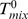 are the equilibrium melting points of PVDF and mixtures, respectively, 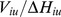
is the heat of fusion of PVDF per unit volume, 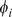 is the volume fraction of PVDF, and B is the interaction energy density between two components. The overall interaction energy density (B) can be obtained from the slope of the plot of 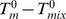 vs. 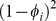. Equation 1 suggests that the parameter B for a ternary blend can be evaluated the same way as for a binary blend.

**Figure 5:**
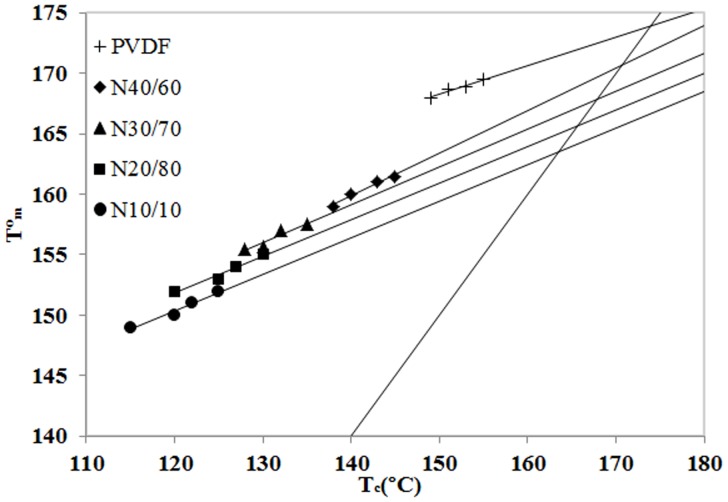
Plots of observed melting temperature 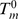 vs. Tc.

The overall interaction parameter B has been evaluated from the equilibrium melting point depression at a given 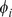. The equilibrium melting points were determined from the Hoffman-Weeks plots, as shown in Figure 5 Table 2 summarizes the equilibrium melting temperature of PVDF in the PVDF/ACM/Clay nanocomposite hybrids, and the data for neat PVDF, PVDF/ACM blends from our earlier work [17]. It is clear that the equilibrium melting temperatures of nanocomposite hybrids are almost the same as the PVDF/ACM blends. This further proves the non-interference of nanoclay in chains crystallization of PVDF.

**Table 2:** equilibrium melting points.

Misciblenanocomposite	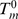	Miscible blend	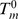
N40/60	171	40/60	171
N30/70	168	30/70	169
N20/80	166	20/80	165
N10/90	163	10/90	158

**Figure 6:**
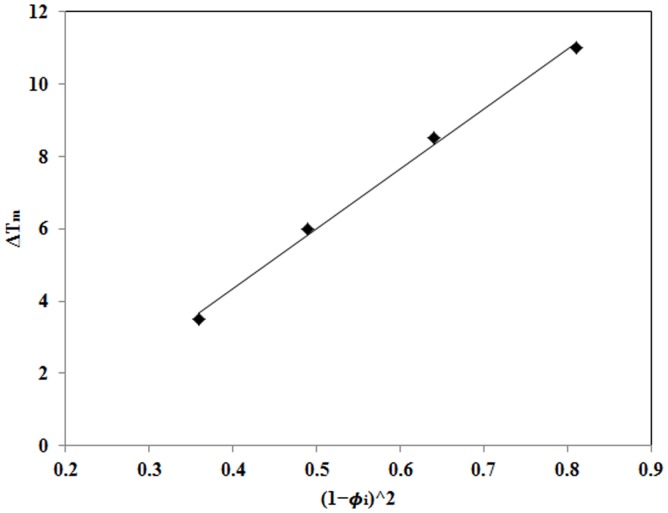
Plot of 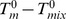 vs. 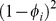.

**Figure 7:**
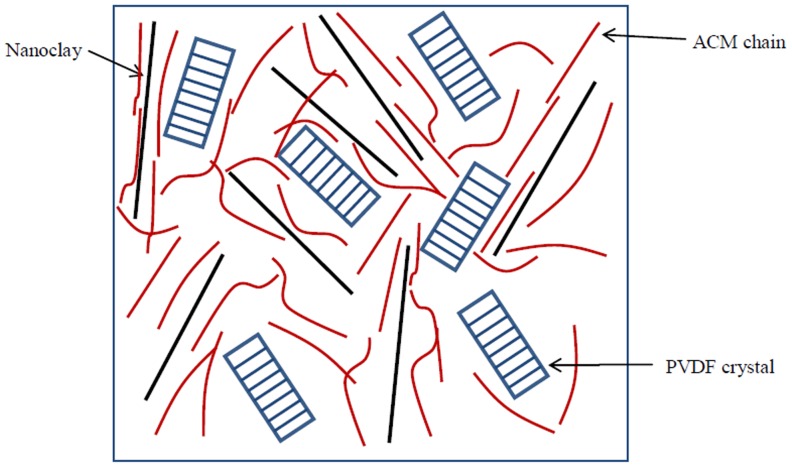
Schematic representation of nanocomposite hybrids demonstrating ACM chains are scattered between PVDF crystals and clay platelets.

Figure 6 shows plots of the equilibrium melting point depression of PVDF versus the square of the volume fraction for the remaining part in the ternary mixture. The data can be fitted by a straight line and its slope determines the overall interaction parameter; B as −2.2 cal/cm^3^. B value of −2 cal/cm^3^ was determined in our previous study [17] for PVDF/ACM blends which is almost the same value as obtained for nanocomposite hybrids. Note that the B value in equation 1 is related to the interaction between crystallizable component with its surroundings. Therefore, the similarity of B values for the nanocomposite hybrid with PVDF/ACM blend is associated with the fact that PVDF chains in both systems interacted with the same environment. In other words, PVDF chains in both systems interact with ACM chains and that nanoclays in hybrid systems are covered by ACM molecules. This hypothesis has been presented schematically in Figure 7. In summary we found that the presence of nanoclay did not affect the formation of α polymorph in miscible nanocomposite hybrids.

## Conclusion

In this study, abnormal formation of α polymorph in a miscible nanocomposite hybrid of PVDF/ACM/Clay has been investigated. WAXD and TEM results proved that clay tactoids formed an intercalated structure in PVDF matrix while miscible nanocomposite hybrids showed individual layers as well as stacks containing parallel and oriented layers with various degrees of intercalation. Miscible nanocomposite hybrids showed similar equilibrium melting points as PVDF/ACM blends, suggesting zero influence of nanoclay in PVDF crystallization.

The Flory-Huggins interaction parameters B value’s between PVDF and its surrounding showed −2.2 cal/cm^3^ and −2 cal/cm^3^ values for PVDF/ACM/Clay miscible nanocomposite hybrids and PVDF/ACM blends, respectively. Therefore similarity of B values for nanocomposite hybrid with PVDF/ACM blend is associated with the fact that nanoclays in hybrid systems are covered by ACM molecules and have no effect on different polymorph formation in PVDF crystallization.
