# Correction: Influence of Miscibility Phenomenon on Crystalline Polymorph Transition in Poly(Vinylidene Fluoride)/Acrylic Rubber/Clay Nanocomposite Hybrid

**DOI:** 10.1371/journal.pone.0099237

**Published:** 2014-05-27

**Authors:** The PLOS ONE Staff

The corresponding author affiliation is incorrect. The correct affiliation for Mohammad Mahdi Abolhasani is: Chemical Engineering Department, University of Kashan, Kashan, Iran.
